# An Assessment of Functional Outcomes and Prognostic Measures Following the Management of Shoulder Dislocations at a Tertiary Care Hospital in the United Kingdom

**DOI:** 10.7759/cureus.72817

**Published:** 2024-11-01

**Authors:** Upamanyu Nath, Roshan Mohindra, Abdullah Bin Sahl, Mohammad Ibrahim, Anand Pillai

**Affiliations:** 1 Trauma and Orthopaedics, Wythenshawe Hospital, Manchester University NHS Foundation Trust, Manchester, GBR; 2 Trauma and Orthopaedics, Royal College of Surgeons in Ireland, Dublin, IRL

**Keywords:** functional score, oxford shoulder instability score, patient reported outcome measures, physiotherapy, shoulder dislocation, simple shoulder test

## Abstract

Background and objective

Shoulder dislocations account for around half of all major joint dislocations in the UK and pose a significant healthcare burden. Management decisions post-reduction procedures involve a range of options, including conservative measures, surgery, and physiotherapy. Patient-reported outcome measures (PROMs), such as the Oxford Shoulder Instability Score (OSIS) and Simple Shoulder Test (SST), have emerged as crucial tools for evaluating treatment outcomes, reflecting a shift towards patient-centred care. This study aimed to evaluate trends related to prognostic factors versus PROMs following the management of shoulder dislocations at a tertiary care centre.

Methods

An observational, retrospective cohort study was conducted at Wythenshawe Hospital, Manchester, involving 70 patients with shoulder instability. Patient demographics, management strategies, and outcomes were evaluated using PROMs, focusing on functional disability assessed by OSIS and SST. Prognostic factors, including age, recurrence, and physiotherapy, were examined to understand their impact on treatment efficacy.

Results

The study included a diverse patient demographic, with both OSIS and SST scores showing a downward trend with increasing age. Physiotherapy correlated with improved SST scores, emphasising its role in rehabilitation. Limited physiotherapy availability and variations in patient-reported data posed challenges in assessing the cohort-wide impact of treatment.

Conclusions

The observed correlation between functional outcomes and patient perceptions underscores the intricate relationship between physical function and subjective experiences, contributing to the nuanced understanding of shoulder dislocation management.

## Introduction

The shoulder joint ranks as the site of the most prevalent large joint dislocation in emergency departments in the UK, constituting 50% of major joint dislocations. As a highly mobile ball and socket synovial joint, its structural complexity, particularly in the glenohumeral joint, makes it susceptible to dislocation and injury. The glenoid fossa's shallow articulation with the humeral head allows extensive motion but sacrifices stability [1-3]. Initial dislocation outcomes vary by age, ranging from recurrent instability due to heightened activity levels in younger patients to rotator cuff injuries in the elderly due to degenerative changes. Given the functional impairment associated with shoulder injuries, effective therapeutic strategies and follow-up care are crucial for the treatment and rehabilitation of these patients [4].

Managing shoulder instability post-reduction procedures involves diverse options, such as conservative measures, surgery, and physiotherapy. Treatment decisions are made based on injury mechanisms, patient preferences, and prognostic factors. A tailored, patient-centred approach, often via shared decision-making, guides management, aiming to enhance outcomes by improving shoulder function, reducing pain and instability, and restoring daily activities [5-7]. Traditionally, the choice of interventions relied on clinical assessments and radiological images. However, a shift towards patient-oriented approaches, specifically using Patient-reported outcome measures (PROMs), is now evident. PROMs, in the form of questionnaires, capture standardised patient perspectives, allowing accurate assessments of treatment outcomes. Numerous PROMs exist, each designed to reflect the patient's subjective assessment of shoulder instability [8-9].

This study focuses on PROMs tailored for evaluating shoulder instability, emphasising the importance of understanding patient priorities in assessing treatment efficacy [8]. It aimed to assess trends related to prognostic factors versus PROMs following the management of shoulder dislocations at a tertiary care centre. We assessed functional disability using the Oxford Shoulder Instability Score (OSIS) and Simple Shoulder Test (SST) and explored nine relevant prognostic factors.

## Materials and methods

Patient selection

This study was an observational, retrospective cohort analysis aimed at evaluating functional outcome measures following therapeutic interventions for shoulder instability. The study included all patients who presented to Wythenshawe Hospital (part of Manchester University NHS Foundation Trust) with shoulder instability between July 2021 and April 2022. The primary objective was to assess the functional outcomes post-intervention.

To identify the relevant patient cohort, we utilized the "Etrauma" electronic patient record system. A search query was coded specifically to identify patients who had been referred to MFT with a diagnosis of shoulder dislocation from July 2021 onwards. This initial search yielded a total of 204 patient records. Subsequently, we applied additional filters to refine the cohort, which resulted in the exclusion of 33 patients due to incomplete data. The remaining patients met the following criteria:

1. They had documented instances of shoulder dislocation.

2. Their patient records were accessible and complete within the "Etrauma" system.

3. They had either been treated for their condition or were under follow-up care at Wythenshawe Hospital during the specified study period.

Further refinement of the cohort was necessary to ensure accurate and complete contact information. A manual search for patient contact details within the ‘Etrauma’ system led to the exclusion of an additional 21 patients due to either a lack of contact information or incorrect telephone numbers. This step was crucial to ensure that only those patients who could be reliably contacted for follow-up were included in the final analysis.

**Figure 1:**
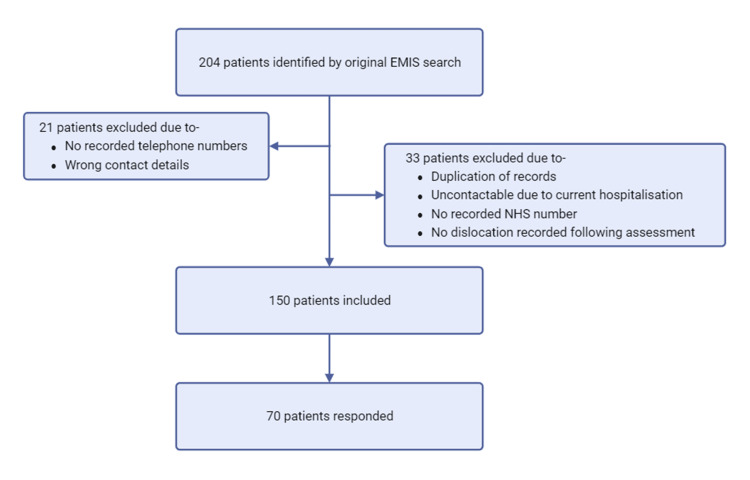
Flow diagram depicting "Etrauma" search to identify suitable patients

Figure 1 shows the process used to identify suitable patients.

All patients included in the study were asked to respond to a series of structured questions designed to gather comprehensive data on their shoulder instability. Specifically, each patient answered 12 questions from the OSIS and 12 questions from the SST questionnaires. These standardized questionnaires are widely used to assess the functional impact of shoulder instability on patients' daily lives. Additionally, a follow-up set of nine questions was administered to identify various prognostic factors that could potentially influence the outcome measures related to their condition. To ensure completeness and accuracy, a manual search of patient records was conducted to obtain any unrecorded responses that had not been previously documented.

The outcome measures that were assessed in this study include:

1. Oxford Shoulder Instability Score

This tool measures the level of instability in the shoulder and its impact on the patient’s quality of life.

2. Simple Shoulder Test

This questionnaire evaluates the functional limitations and symptoms associated with shoulder instability.

3. Number of Recurrent Dislocations

This measure tracks the frequency of dislocations that patients experienced over the study period.

In addition to the primary outcome measures, several prognostic factors were examined via a researcher-administered questionnaire to better understand the variables that might influence the outcome of shoulder instability. These factors include:

1. Initial Versus Recurrent Dislocation

Patients were asked whether their shoulder dislocation was the first occurrence of its kind or a recurrence of a previous dislocation.

2. Activity at the Time of Dislocation

For recurrent dislocations, the study sought to determine what activity led to the dislocation. Patients were asked if the dislocation occurred while at home, at work, during sports activities, or while at the gym.

3. Dominant Arm Involvement

It was recorded whether the dislocated shoulder was the dominant arm of the patient.

4. Mechanism of Injury

Patients were asked to describe the mechanism of the shoulder dislocation, distinguishing between traumatic and atraumatic causes.

5. Management of Dislocation

Information was collected on whether the shoulder dislocation was managed conservatively (non-surgically) or through surgical intervention.

6. Physiotherapy

The study assessed whether the patients received physiotherapy as part of their treatment plan.

7. Occupation

Data were gathered on the patient’s occupation to evaluate potential occupational impacts on their shoulder condition.

8. Time Off From Work

Patients were asked how long they were advised to take time off from work and the actual duration they took.

9. Level of Activity Prior to Dislocation

To assess the pre-existing level of physical activity, patients were categorized based on their exercise habits

 a. Inactive: Less than 30 minutes of exercise per week

 b. Active: Between 30 and 149 minutes of exercise per week

 c. Very active: 150 minutes or more of exercise per week

Literature review

Recurrent Instability and Its Impact

Recurrent instability is a common post-initial shoulder dislocation, with about 90% occurring within two years [4]. Factors like age, post-dislocation pathology, and activity levels influence the risk of recurrence, with younger individuals having a higher risk [5]. Males under 20 years old may have a 72% chance of recurrent instability [4]. This recurrence increases the risk of arthropathy, fear of dislocation, and poorer surgical outcomes, emphasizing the importance of preventive strategies through optimal initial management.

Conservative Management

For patients over 40 with reduced recurrence risk, conservative approaches like immobilization and physiotherapy are suitable. Physiotherapy addresses key areas such as restoring rotator cuff strength, improving range of motion, optimizing flexibility, and enhancing functional strength. Despite these efforts, recurrent instability occurs in 52% of cases [5].

Surgical Management

The decision to perform surgery, complex and based on factors like age and dislocation frequency, is usually taken after the failure of conservative treatment. However, surgery carries risks, and conflicting evidence from randomized controlled trials has led to diverse approaches. Decision-making involves a shared discussion between patients and doctors [4,5,7].

Patient-Reported Outcome Measures (PROMs)

Recurrent shoulder instability impacts patients' quality of life, causing anxiety and loss of confidence [10]. Recognizing patient-surgeon priorities, a patient-focused approach using PROMs has gained significance. These measures capture both subjective patient experiences and objective outcomes. Commonly used shoulder-specific questionnaires include OSIS and SST.

Oxford Shoulder Instability Score (OSIS): OSIS, a 12-item condition-specific questionnaire, assesses surgical and non-surgical outcomes in shoulder instability. Responses are rated on a Likert scale, with the total score ranging from 0 to 48, categorizing outcomes as poor, fair, good, or excellent (Appendices) [11].

Simple Shoulder Test (SST): SST is a yes/no questionnaire with 12 items addressing function-related pain, strength, and range of motion. Scores range from 0 to 12, reflecting the worst to best outcomes (Appendices) [12].

## Results

Patient demographics

**Figure 2:**
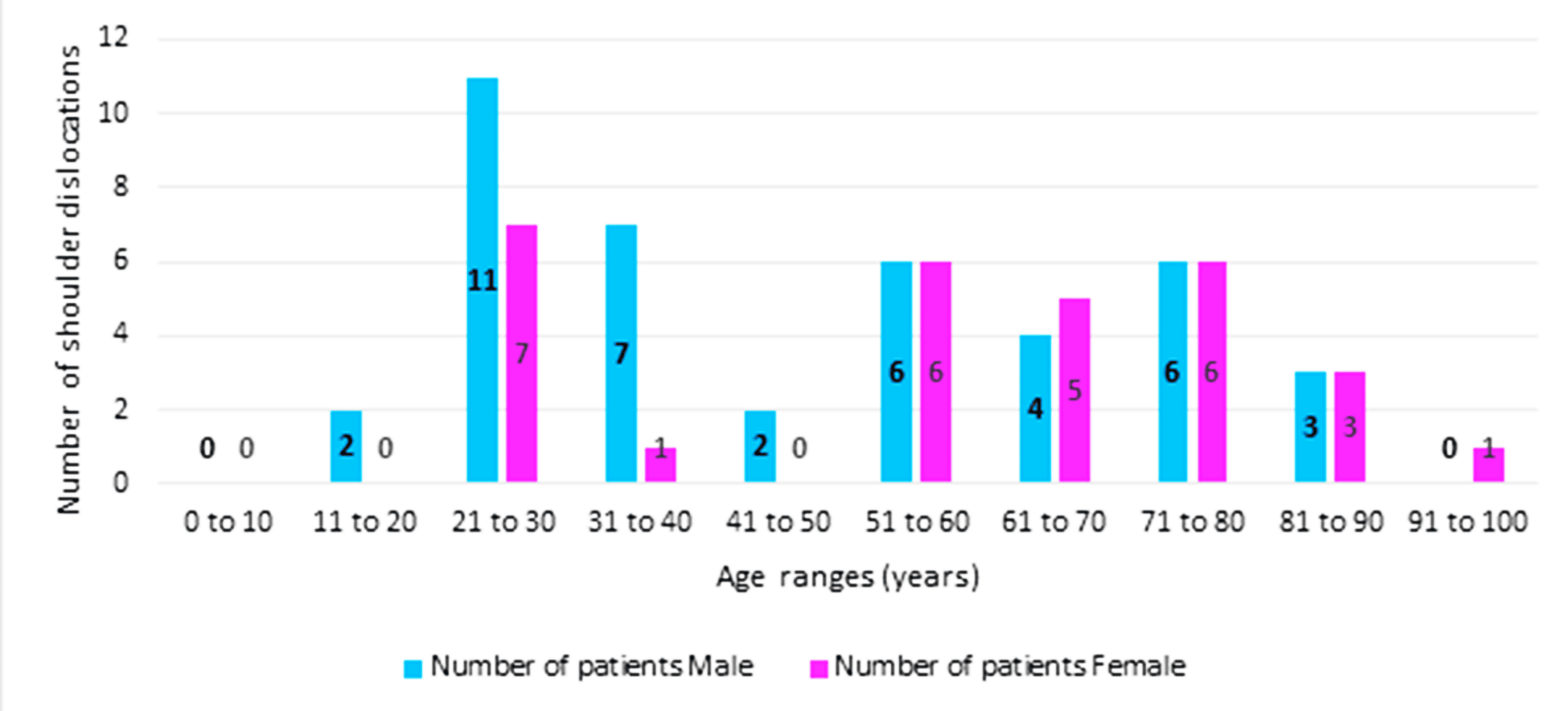
Overall patient demographics

As seen in Figure 2, 70 patient responses were collected for analysis; 29 patients were female and 41 male, with ages ranging from 16 to 93 years.

Dominant versus non-dominant

Of the 70 dislocations, 48 were of the dominant shoulder and 22 of the non-dominant shoulder. 

First-time versus recurrent dislocation

55 out of 70 were first-time dislocations and 15 were recurrent. Of the recurrent cases, nine occurred while carrying out routine activities of daily living at home, one occurred while working and five resulted from a sporting injury. 

Management of dislocation

Of note, 61 out of 70 patients were managed with a polysling or arm sling and nine underwent shoulder surgery. 

Follow-up

In the cohort, 36 out of the 70 patients were followed up within two weeks at a Shoulder and Elbow (S&E) Clinic; 23 patients were discharged with advice and physiotherapy referral while 11 patients were discharged with advice alone. 

Physiotherapy

In total, 30 patients were referred for physiotherapy, including those referred following their treatment at the S&E clinic and, those discharged with advice and physiotherapy referral. Of these, 21 patients reported having physiotherapy treatment, and two patients discharged with advice alone underwent therapy privately. 

Time off from work

Twenty-seven out of 70 patients reported receiving advice to take time off work due to injury. Out of the rest, 21 were not advised, 16 were retired and hence advice did not apply, and six were unsure. Of the 27 patients who received advice, 14 reported to have taken the exact time recommended, five took more time off and eight took less time than recommended. 

Level of activity

Patients were asked about their activity levels before the shoulder dislocation. Nineteen patients reported being very active (>150 minutes of exercise), 43 were active (30-149 minutes) and eight were inactive (<30 minutes). 

Oxford Shoulder Instability Score (OSIS)

**Figure 3:**
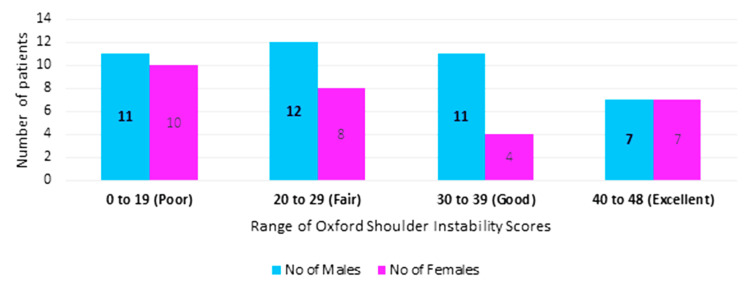
Patient demographics for OSIS OSIS: Oxford Shoulder Instability Score

As shown in Figure 3, OSIS ranged from a minimum of 5 out of 48 to a maximum score of 48 out of 48. The mean cohort score was 28 and the mode was 18; 21 out of 70 scored a poor score of 0 to 19 (11 males and 10 females); 20 out of 70 scored a fair score of 20 to 29 (12 males and eight females); 15 out of 70 scored a good score of 30 to 39 (11 males and four females); 14 out of 70 scored an excellent score of 40 to 48 (seven males and seven females).

OSIS Versus Age

**Figure 4:**
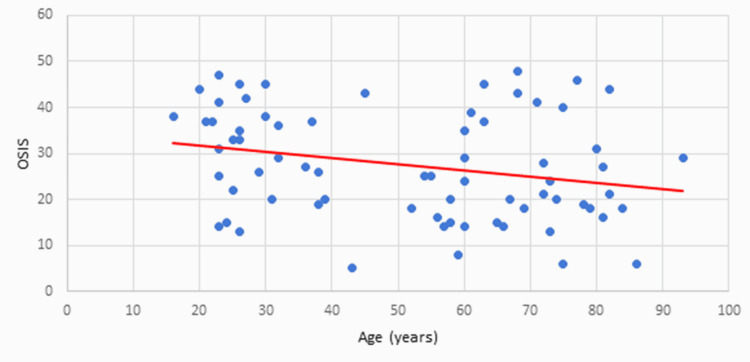
OSIS versus age OSIS: Oxford Shoulder Instability Score

As evidenced in Figure 4, OSIS exhibited a downward trend with increasing age; 42 out of 70 patients were aged over 40 years, and their mean OSIS was 25 out of 48; 28 patients were aged under 40 years, and their mean OSIS was 31 out of 48. 

OSIS and Physiotherapy

Twenty-three patients who undertook physiotherapy had OSIS ranging from 13 to 45 out of 48. The mean was 28, and the mode 37. The scores of the 47 patients who did not have physiotherapy ranged from 5 to 48, with a mean of 27 and a mode of 15. 

Simple Shoulder Test (SST)

**Figure 5:**
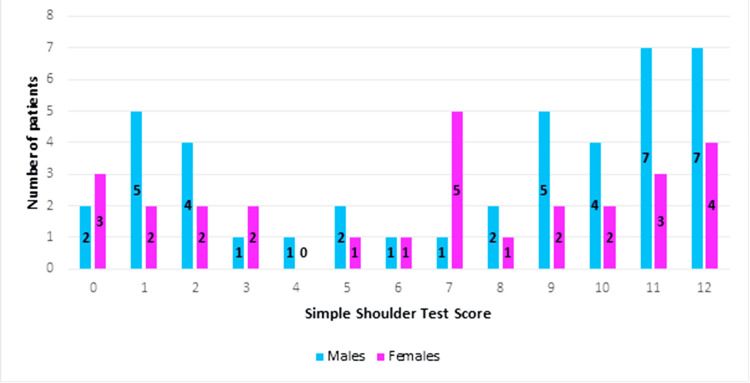
Patient demographics for SST SST: Simple Shoulder Test

Figure 5 shows the demographics for each score in SST according to the gender of the participants.

SST scores ranged from 0 to 12 points, with a mean of 7 and a mode of 12. 

SST Versus Age

**Figure 6:**
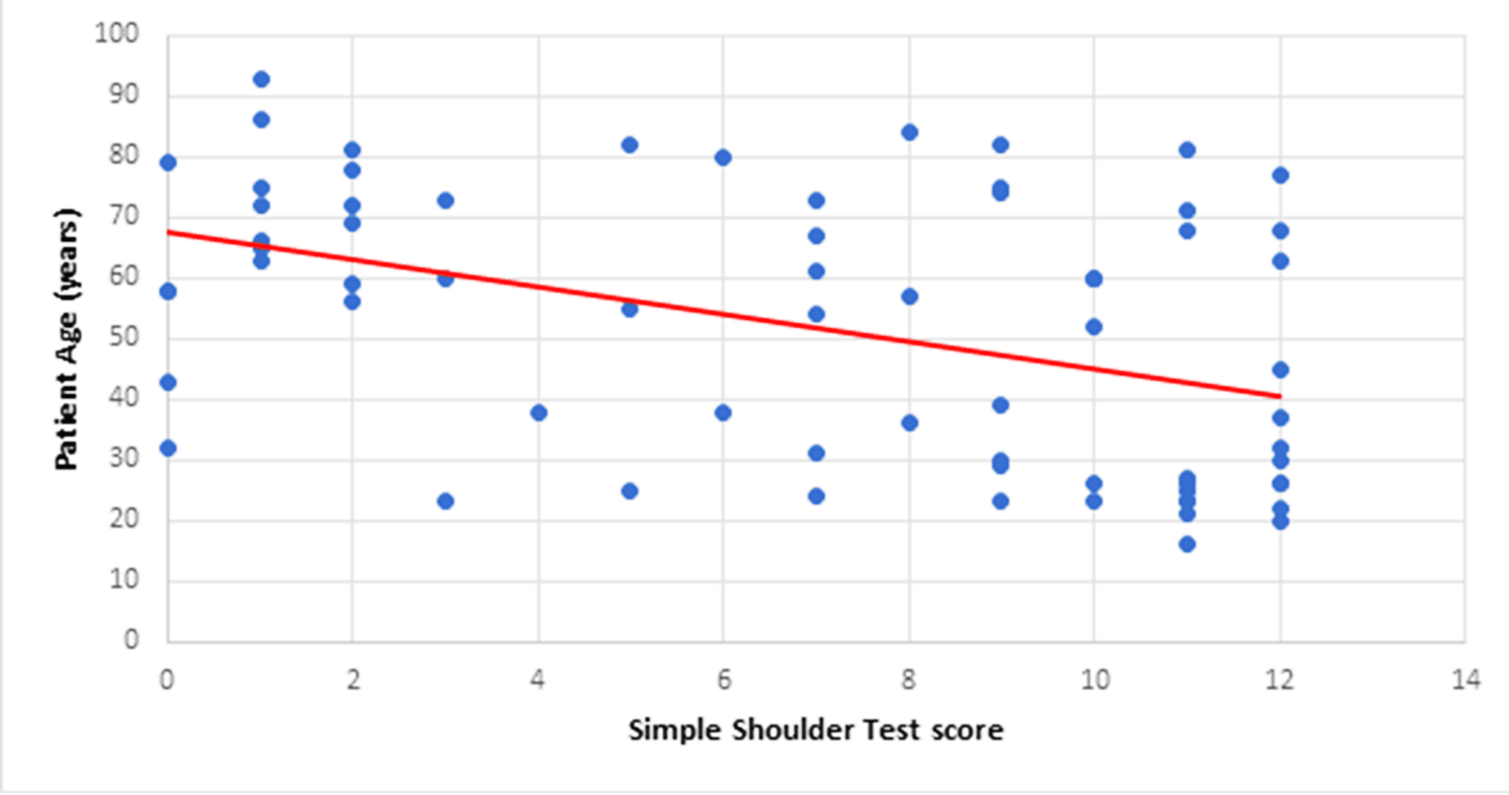
SST versus age SST: Simple Shoulder Test

The mean SST score for patients below 40 years old was 9 out of 12 while it was 6 for those above 40 years old. As illustrated in Figure 6, SST showed a downward trend with increasing age.

*SST and Physiotherapy* 

SST scores ranged from 1 to 12 points in those who had physiotherapy. The mean value was 9 and the mode was 10. The score for those without physiotherapy ranged from 0 to 12, with a mean score of 6 and a mode of 11. 

Simple Shoulder Test (SST) versus Oxford Shoulder Instability Score (OSIS)

**Figure 7:**
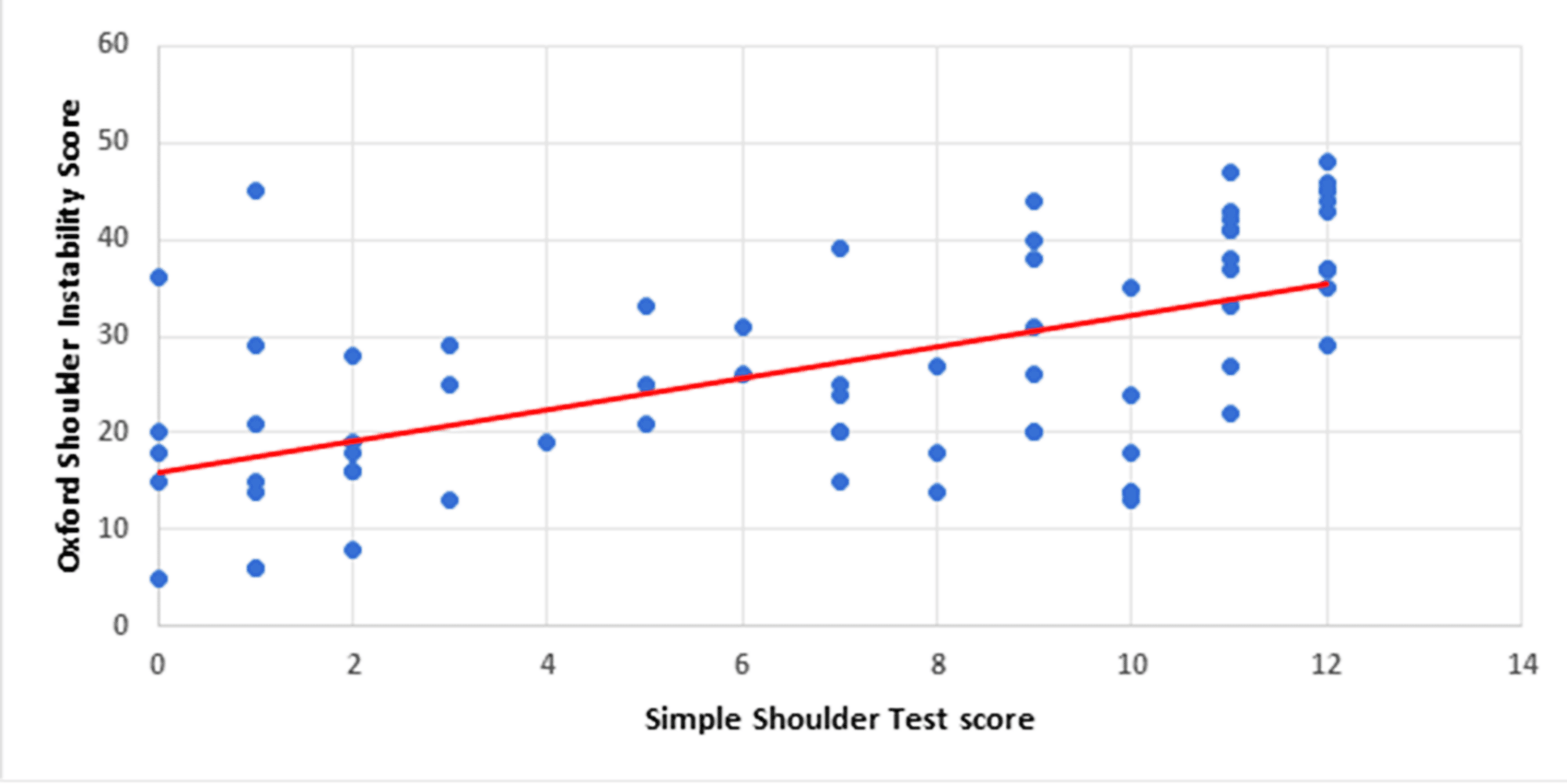
Oxford Shoulder Instability Score versus Simple Shoulder Test Score

SST and OSIS scores were compared among the same patients to assess their function against injury anxiety and perceptions. The trend depicted in Figure 7 suggests that better SST scores correlated with better OSIS and vice-versa.

Additional prognostic factors

Other prognostic parameters that may influence functional outcome scores were documented and analysed but no significant and replicable trends were observed.

## Discussion

Factors influencing outcome measures

Age

Our analysis of specific patient factors influencing OSIS and SST scores revealed a wide age range (16-93 years), with the 21-30 age group experiencing the most dislocations (n=18). However, over 40-year-olds had more dislocations (n=42) than those under 40 (n=28). Adjusting OSIS for age showed a minimal mean change from 28 to 25 for those over 40 years and an increase to 31 for those under 40, suggesting slightly better scores for younger patients. Similar shifts were observed in the mean values for SST, implying younger patients had a higher range of motion and strength. Unequal distribution in age ranges may skew the data on account of our sample size.

Physiotherapy

When comparing mean OSIS values, patients with and without rehabilitation had means of 28 and 27, respectively, with a mode shift of 37 in the physio group and 15 in the control group. However, the sample size difference (23 with physio, 47 without) complicates the interpretation of this finding. SST scores showed improvement from 7 to 9 in the rehabilitation group and a decrease to 6 in the non-therapy group, suggesting better strength and range of motion outcomes with rehabilitation. Conflicting evidence found in the literature on physiotherapy efficacy emphasises the need to conduct studies involving larger sample sizes for accurate assessments [4,5,13].

Resource Availability

The National Institute for Health and Care Excellence (NICE) guidelines highlight the significance of early mobilisation and physiotherapy referral for acute shoulder dislocations, with recommended physiotherapy courses lasting 4-12 weeks. Other studies and BOA/BESS algorithms further advocate physiotherapy for atraumatic shoulder injuries [4,14,15]. However, in this study, only 30 out of 70 patients received physiotherapy, with 21 accepting treatment, and two organising private sessions independently.

Reported measures indicate that those undergoing physiotherapy exhibited better SST scores than those who did not. The case-specific nature of each referral, coupled with limited resources, poses challenges in assessing the cohort-wide impact of treatment. Patient feedback revealed a lack of written advice or leaflets post-dislocation, hindering recall of specifics on recovery. Access to detailed information and specific advice was primarily available to those attending the S&E clinic, highlighting the need for improved patient awareness regarding physiotherapy referral options.

However, the study's reliance on patient-reported data and variations in physiotherapy regimens limit the standardisation of therapeutic outcomes. Addressing these challenges is crucial for optimising post-shoulder dislocation care.

Limitations

This study heavily relies on accurate coding in the "Etrauma" search for patient identification within the target population. Assumptions about contemporaneous and accurate patient records may have introduced some errors due to inadequate documentation and patient recall. Limited patient response meant that we had a relatively small sample size, which may not have been sufficient to represent overall functional outcomes. Hence, we recommend studies with a larger sample size. Moreover, since the study was retrospective in nature, it lacked baseline data, hindering a comprehensive assessment of intervention effects on outcomes. No initial scores were recorded at the time of intervention, preventing a full evaluation of improvements. Future adaptations, such as prospective cohort tracking or repeating these scores at a later time, could address these limitations.

Additionally, the absence of functional outcome data for the asymptomatic population raises uncertainties as to whether improvements align with average scores. Patient attitudes and expectations may differ based on baseline scores, influencing perceptions of intervention success. Given these concerns, we advocate for a more robust design in future studies [16].

## Conclusions

This dataset provides a nuanced understanding of the demographics, management strategies, and outcomes associated with shoulder dislocations. The emphasis on physiotherapy highlights its importance in the rehabilitation process. The observed correlation between functional outcomes and perceptions of shoulder stability underscores the intricate relationship between physical function and patients' subjective experiences, where better outcomes were associated with younger age. SST, which assesses strength and mobility, showed better results than OSIS. Further research and exploration of these findings may help devise enhanced strategies for the management and rehabilitation of individuals with shoulder dislocations.

## Figures and Tables

**Figure 8:**
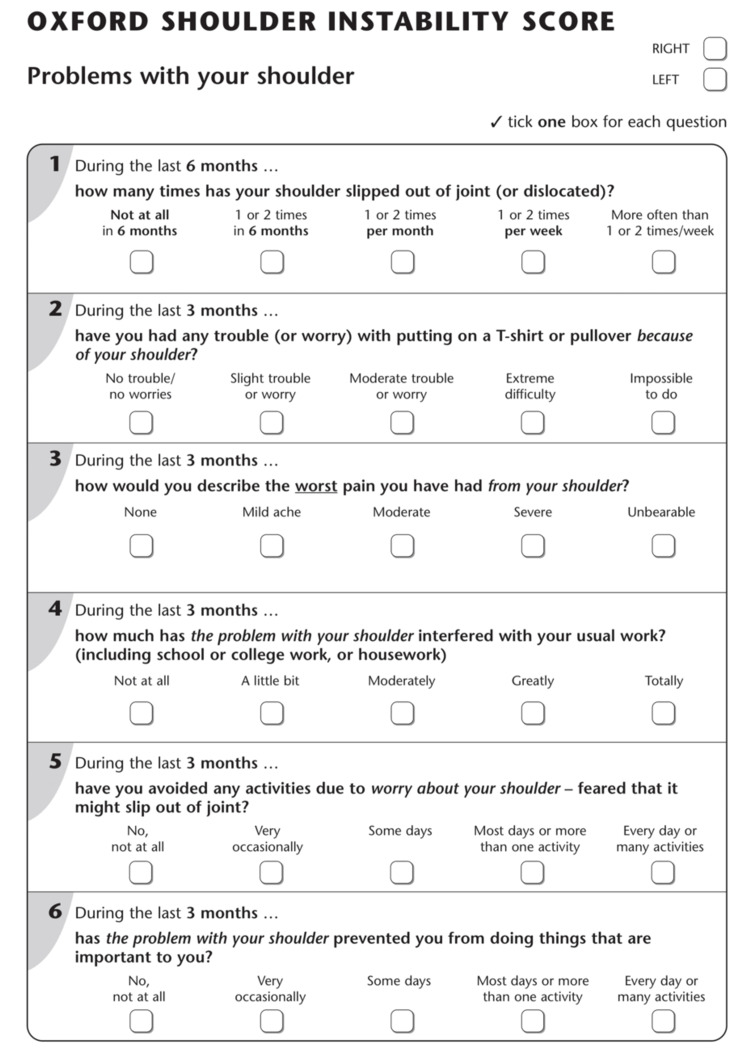
Oxford Shoulder Instability Score questionnaire (1/2)

**Figure 9:**
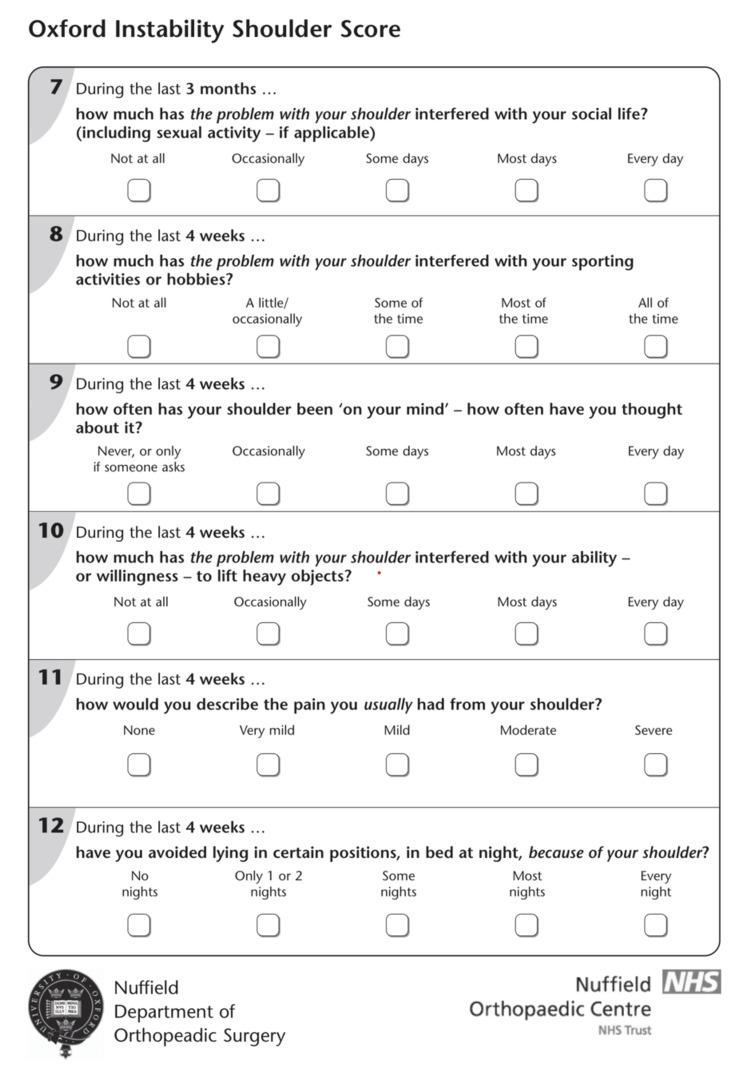
Oxford Shoulder Instability Score questionnaire (2/2)

**Figure 10:**
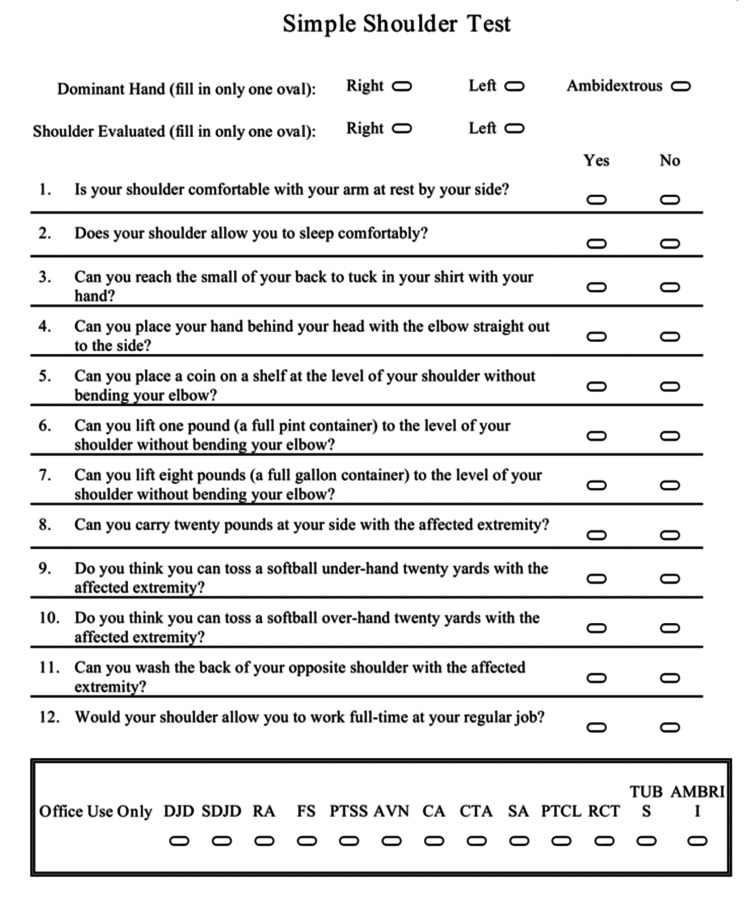
Simple Shoulder Test questionnaire
